# Some Verifications of Similia

**Published:** 1891-03

**Authors:** D. H. Dean

**Affiliations:** Columbus, Ind.


### SOME VERIFICATIONS OF SIMILIA.

D. H. Dean, M. D., Columbus, Ind.

(Read at the Twenty-fourth Annual Session of the Indiana Institute of Homœopathy, Indianapolis, May 15th, 1890.)

My paper will necessarily be a random one, as you may know from the subject.

Every science must have some fixed laws upon which it is based. In order that we may know that it is certainly a science these laws must be clearly demonstrated. The cardinal principle of our science of therapeutics is, as every homœopath knows, that great law enunciated by Hahnemann almost a century ago, *Similia similibus curantur.* Every day of practice is only adding to the certainty of this law, and the time is not far distant when we may proclaim its universality.

In this paper I wish to give briefly some of my experience with a few drugs, showing the practicality of similia.

My first drug is the action of Rhus-tox in lumbago. Case: A lady, aged fifty, had been suffering severely with lumbago when she applied to me for relief, with symptoms as follow:

Unable to straighten her back and must walk in stooped position. Slight twist in back caused excruciating pain. Unable to remain long in one position and some relief by moving about the room. She was a lady who did considerable lifting, and had, as she thought, probably overstrained herself. She also stated that damp weather caused aggravation of her symptoms. All these symptoms led me to the certain choice of Rhus, which I gave in the 3x dilution. She reported that she felt relief after the first dose, and in less than three days every symptom had disappeared. This was very prompt action, and of course was due to the fact that the drug was well indicated.

The old-school doctors are gradually "getting on to" the therapeutic values of this drug, and Dr. John Aulde, of Philadelphia, spends a good deal of time detailing some conditions calling for this drug. He says it is impossible for him to tell just when it should be given and when not in sciatica, etc. We would just tell him to make a little deeper study than he has done of our materia medica. He prefers one-half drop doses. Now this gentleman would have his colleagues believe that these values of this drug have been hitherto unknown, and that it was reserved for him to make the discoveries and hold them up as triumphs of allopathy. There is very little doubt his discoveries were like mine, made from the homœopathic materia medica, and the same may be said of his much extolled Arsenite of Copper, which he gives—shades of Cæsar!—in doses of one-thirty-two hundredth grain for cholera morbus, etc. Instead of explaining it by the law of similars, he calls it one of the enigmas of medicine and goes on. Very scientific indeed. But let this peculation go on as it's only the better for the patient.

My next remedy is China. The case was that of a gentleman aged forty who had been troubled with a diarrhœa for three weeks. Just previous to his taking the diarrhœa he had been traveling about two weeks, eating a great deal of fruit and otherwise careless with his diet. After returning home he tried dieting himself and took several simple home remedies, but to no purpose. The stools were aggravated by eating; some colic previous to going to stool; stools blackish in color and containing undigested matter; a good deal of debility. I prescribed

China, ten drops tincture in one-half glass of water. This produced marked aggravation. I then put one teaspoonful of this solution in one-half glass of water, and he felt relief from the first dose and very soon was entirely well. He afterward told me that what surprised him was that this checking of the diarrhœa was not followed by constipation and said he was a convert to that method of healing.

The next case I wish to report is a case of orchitis, where Pulsatilla proved the curative remedy. Case was a young man who contracted gonorrhœa three months previously to the present trouble. All acute symptoms had of course subsided, but there remained a gleet and some slight soreness in two or three spots in urethral track, all no doubt due to badly managed treatment of the primary trouble. He was a moulder by trade and strained himself by overlifting one half day. This was followed by much soreness and swelling of testicles. I prescribed Arn.$^{2c}$, and told him to do only light work. The trouble was promptly relieved, but now unknown to me he began the use of a patent nostrum, warranted a sure cure for gleet when used as an injection. The result was a cessation of the discharge and a return of the orchitis in a much more aggravated form than previously. There was drawing through spermatic cords, tearing pains in testicles, and extreme sensitiveness to slightest touch. All these symptoms pointed unerringly to Pulsatilla, which I prescribed, ten drops tincture in one-half glass of water—teaspoonful four times a day—also putting on a suspensory bandage. This in a very few days completely relieved him and there has been no return of the orchitis since, although he has kept right on at his work moulding. I might add that he had gone to a regular physician, who prescribed a large bottle of nauseating stuff and told him to go to bed. The thought of swallowing this shot-gun mixture and of losing the two dollars and fifty cents a day he was making turned him against the regular, and the result was the Pulsatilla treatment and a few dollars more in his inside pocket.

This is another drug the allopaths are stumbling on to for these cases, and giving themselves great credit for their new discoveries.

Dr. Tucker, of Texas, speaks of its great value in orchitis in two-drop doses. The *Medical World* says better results are obtained with one-drop doses. Homœopaths for almost a century have had fine results where it is indicated in fractional drop doses.

I might detail many more cases verifying similia, but it would just be a repetition of what every homœopath has witnessed over and over again in his daily practice. However, I believe there is a tendency to laziness in a great many of us, and hence a carelessness in prescribing and a doubtfulness in results. Then Homœopathy is censured when only the practitioner is at fault. I believe Homœopathy has indeed very few faults, and that if we are constantly awake to our work and adhere strictly to its principles we may always expect prompt results. We are too liable to fall into a rut and prescribe on general principles or empirically like the ancient school. There is too much prescribing Nux-vomica for a headache, stomach trouble, or constipation, or of Aconite for every fever, or of Belladonna for all sore throats. Just so long as we adhere to this method of prescribing we are nothing but empirics and need not expect to achieve any greater success than our friends of the old school; in fact, not so great. If we can prescribe no more accurately than for the name of a disease, as to a greater or less extent many of us are unconsciously doing, we had better take down our banner and no longer pretend to be what we are not—disciples of Hahnemann. The fact is it's no easy task to be a successful prescriber according to similia. It requires both a close and careful study of the case before you and the materia medica. It is a little more difficult than prescribing Quinine for every case of malaria, Calomel for constipation, or Opium for diarrhœa, or as, in the case of homœopaths, Nux-vom. for all stomach troubles, Aconite for all fevers, etc. In the language of the veteran homœopath, Jahr: "Our art is and will always remain *an art of observation* which has for its object not only to investigate the effect of drugs on persons in health but likewise to examine the condition of the patient in every individual case in all its aspects, and, after administering the proper remedy, to examine him again, in order to find out what further course the disease will take."

